# Is it possible for artificial intelligence (AI) to develop a personality?

**DOI:** 10.1192/j.eurpsy.2025.590

**Published:** 2025-08-26

**Authors:** E. Molchanova

**Affiliations:** Psychology, American University in Central Asia, Bishkek, Kyrgyzstan

## Abstract

**Introduction:**

The development of artificial intelligence (AI) has led to significant advancements in various fields, including mental health applications. As AI technologies like ChatGPT continue to evolve, questions have arisen about whether AI can eventually develop a true personality, and what implications this might have for fields such as psychology and psychiatry. Isaac Asimov’s ideas about AI and the Turing test have gained renewed attention, yet these frameworks do not address the core psychological components such as empathy, emotion, and personal interaction—key elements in therapeutic settings.

**Objectives:**

This article explores whether AI could develop a personality and replace human therapists in psychological counseling and psychiatry. Specifically, it aims to evaluate AI’s current capabilities in providing emotional and psychological support and to address whether AI can evolve to meet therapeutic practice’s deeper, human-centered requirements.

**Methods:**

The analysis is based on reviewing current AI applications in mental health, such as AI-based therapy platforms for post-traumatic stress disorder (PTSD), which provide symptom management tools and promote adaptive coping strategies. These applications were compared to the human therapist’s role, focusing on emotional interaction, empathy, and the therapeutic relationship. Additionally, the philosophical and psychiatric aspects of personality formation in both humans and AI were examined.

**Results:**

AI systems have made progress in simulating therapeutic techniques, providing guidance, and mimicking emotional responses. They can support symptom relief and enhance coping strategies, especially in areas where human therapists are scarce. However, AI’s ability to engage with deeper aspects of the therapeutic process, such as emotional empathy and personal connection, remains limited. AI lacks subjective experience, emotional depth, and self-awareness—essential factors for forming a genuine personality.

**Conclusions:**

While AI has the potential to augment clinical practice, it cannot replace the human element in therapy. The development of AI-based tools is valuable for symptom management, but psychotherapy is inherently rooted in human connection, intuition, and emotional engagement—qualities AI does not possess. For AI to truly replace human therapists or develop a personality, significant advancements in consciousness and emotional cognition would be required, which remain speculative at this stage. Thus, AI will likely continue to serve as a supportive tool rather than a replacement for human therapists in the foreseeable future.

**Disclosure of Interest:**

None Declared

